# Laparoscopic single-incision gastric bypass: initial experience, technique and short-term outcomes

**DOI:** 10.1186/s13022-015-0016-z

**Published:** 2015-10-15

**Authors:** Ivan Alberto Zepeda Mejia, Tomasz Rogula

**Affiliations:** Hospital de Clinicas de Porto Alegre, Universidade Federal do Rio Grande do Sul, Porto Alegre, Brazil; Cleveland Clinic, Bariatric and Metabolic Institute, 9500 Euclid Ave, M66-06, Cleveland, OH 44118 USA

**Keywords:** Single incision laparoscopic surgery, Single incision gastric bypass, Bariatric surgery, Post-operative Pain, Cosmesis, Morbid obesity

## Abstract

**Background:**

Single incision laparoscopic surgery (SILS) research has been limited. The aim of this study is to describe our technique and to evaluate the short term outcomes and efficacy of SILS Roux-en-Y gastric bypass (RYGB) in a selected group of patients in a single center.

**Methods:**

From March 2012 to January 2013, a total of fourteen patients underwent SILS RYGB using a single vertical 2.5–3 cm intra-umbilical incision, 3-ports placed trans-fascially, and a liver suspension technique in Cleveland Clinic’s Bariatric & Metabolic Institute, in Cleveland, Ohio, USA. Patient selection, short-term outcomes and technical issues were retrospectively viewed in this study.

**Results:**

A total of 14 morbid obese patients (12 women and 2 men; mean age, 46 years). Mean operative time was 196 (range 131–265) min. Mean weight at surgery was 113 (range 91–135) kg. One patient required placement of one additional port (7 %). No conversions to conventional laparoscopic surgery (CLS) or open surgery was needed. The estimated blood loss was 40 (range 20–100) ml. In terms of pain control, the frequency of patient controlled analgesia had a mean use of 21 times in postoperative day 0 (POD), 37 times in POD1 and 13 times in POD2. Pain score (assessed by visual analogue scale) had a median score of 6.9 in POD0, 5.2 In POD1 and 3.8 in POD2. Weight loss was approximately 7.25 lb. (±4.5) after first postoperative visit, 28.9 lb. (±11.86) after 1 month and 45.4 lb. (±15.4) after 4 months. No patients required re-operation or readmission during the 90 days after surgery.

**Conclusion:**

Single incision is feasible, safe and reproducible technique used as an access to complex surgeries like gastric bypass in carefully selected patients. Results in short-term outcomes are comparable to those observed in literature. Some potential benefits include less postoperative pain, improved cosmesis, and patient satisfaction. Randomized trials involving larger patient series with a longer follow-up and larger cohort studies and/or systematic reviews will be necessary to assess the extent of the benefits and limitations of SILS in bariatric surgery.

## Background

Bariatric surgery has shown to be the best treatment for morbid obesity. Laparoscopic bariatric surgery has several proven advantages when compared to open approach, including decreased postoperative pain, less postoperative complications, shorter hospital stay, faster recovery, and better cosmesis [1]. In opposite to the revolutionary change from open surgery to laparoscopic bariatric surgery, single-incision laparoscopic surgery (SILS) has questionable benefits when compared to laparoscopic bariatric surgery. This procedure is still under evaluation for its utility in the field of bariatric surgery. Developments in surgical instruments and maturation in surgical technique have made it more available and appealing, especially in short follow-up. Better cosmesis, potential less postoperative pain and shorter hospital stay are potential advantages of SILS over conventional laparoscopic bariatric surgery in some studies, however there is no convincing clinical trial proving this [2]. Current trends show that most of the research published about SILS for bariatric surgery is dated from 2 to 3 years ago. SILS overall interest seems to be less in the past years, possibly because of the complexity of the procedure. Some reports have shown that in experienced hands, SILS can be a feasible and safe procedure in bariatric surgery [3]. In this study, we present our experience with single incision Roux-en-Y gastric bypass in 14 patients, focusing on describing a simplified surgical technique.

## Methods

### Patients

The study group was comprised of 14 patients who underwent single incision laparoscopic gastric bypass at the Cleveland Clinic Bariatric and Metabolic Institute between March 2012 and February 2013. All patients met NIH criteria for bariatric surgery. They were selected at the preoperative visit based on physical exam and body habitus and some of the major inclusion criteria of concern were BMI < 50 kg/m^2^, abdominal wall fat distribution and absence of prior abdominal surgery history (abdominal scars could affect the cosmetic benefit). Also, patients with thick abdominal wall and those with tall stature were not considered for SILS. For the remaining 14 patients, a detailed informed verbal and written consent was obtained.

### Outcomes measured

Operative time, estimated blood loss, need for conversion, length of hospital stay, complications, reoperation, short-term weight changes and wound satisfaction perioperative and 90-day postoperative were the outcomes measured in this study.

We used the visual analogue scale (VAS) to subjectively evaluate pain. An 11-point numeric scale with 0 representing “no pain” and 10 representing “worst pain imaginable” [4]. First evaluation of pain was 6–8 h after surgery. Formerly, they were assessed daily during the duration of their hospital stay, via telephone 3–5 days after discharge and finally during the first follow-up visit 7–10 days after discharge. Patient controlled analgesia (PCA) pump activations were used to evaluate objective pain. The amount of times used in postoperative day (POD) 0, 1, and 2 were computed. We measured the oral pain medication use by the numbers of doses of oral liquid narcotic taken by the patients on POD 1 and POD 2 and the proportion of patients who used oral narcotics after hospital discharge. Postoperative nausea and vomiting was assessed by counting the use of standard anti-emetics (Ondansetron and/or Scopolamine topical patch).

Short-term weight changes were assessed at 1 and 4 months after surgery. Every patient was asked for their grade of cosmetic wound satisfaction in a scale of 1–3. Objectively the patients were evaluated for infections, presence of hernias, seromas, wound dehiscence, and size of scar.

### Surgical procedure (SILS)

#### Ports and Instruments

One 12-mm trocar with Optiview technology (Ethicon), two 5 mm port (Covidien), a 5-mm 45-degree angled camera, regular inline graspers, Endo-Stitch (Covidien) suturing devices and when possible, we utilize a cordless ultrasonic dissection device (Sonicision, Covidien) with a long shaft. Powered articulating staplers (Ethicon) with a linear load of 60-mm white, blue or green cartridges are used depending on transected tissue thickness.

#### Accessing the abdomen

**Fig. 1:**
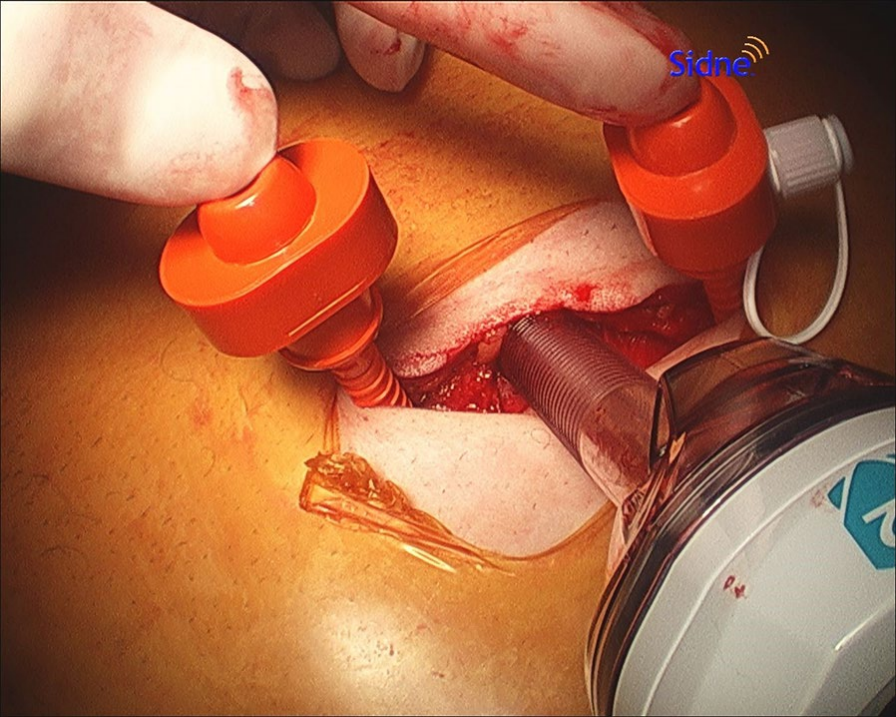
Port placement for single incision laparoscopic gastric bypass

A vertical 2–3.5 cm skin incision is made starting slightly off and in the cusp of the umbilicus, proceeding towards the upper umbilical edge, exceeding it as needed. A 2–3 cm space underneath the subcutaneous fat and over the abdominal fascia is dissected for port placement. Pneumoperitoneum (12–15 mmHg) is created with the use of a veress needle inserted through the lower-middle part of the exposed fascia. A 12-mm Optiview trocar is then inserted, centrally and slightly to the lower-middle part of the exposed fascia, under direct visualization with a zero degree laparoscope. Next, a triangle with approximately 2 cm sides is created by blindly inserting two 5-mm ports laterally and superior to the 12-mm port towards the subcutaneous pocket (Fig. 1).

#### Retraction of Liver

**Fig. 2:**
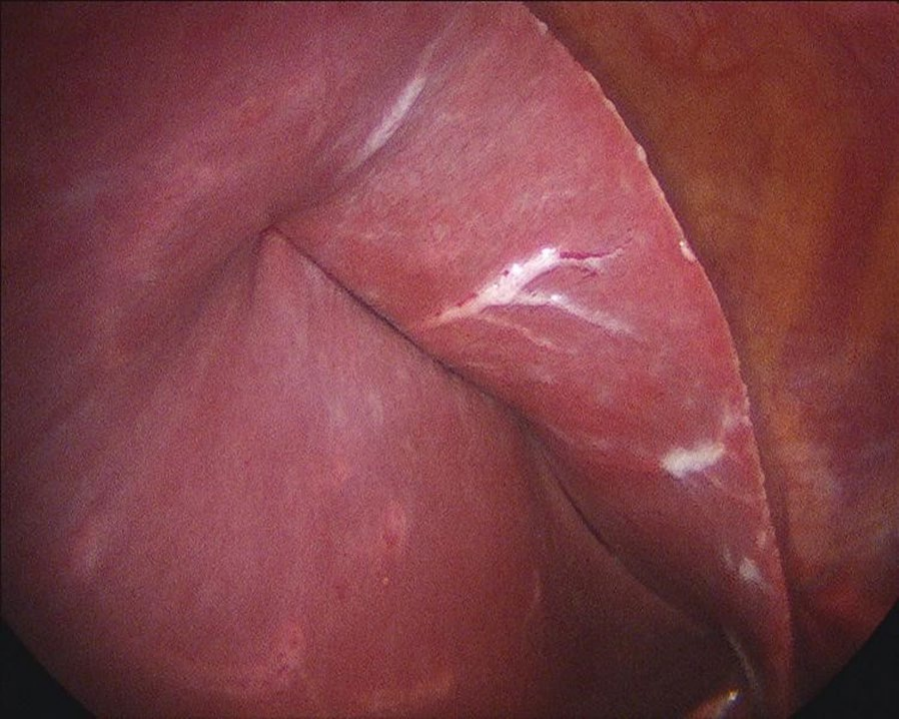
Technique of liver retraction with a stitch passed through the left lobe of liver

**Fig. 3:**
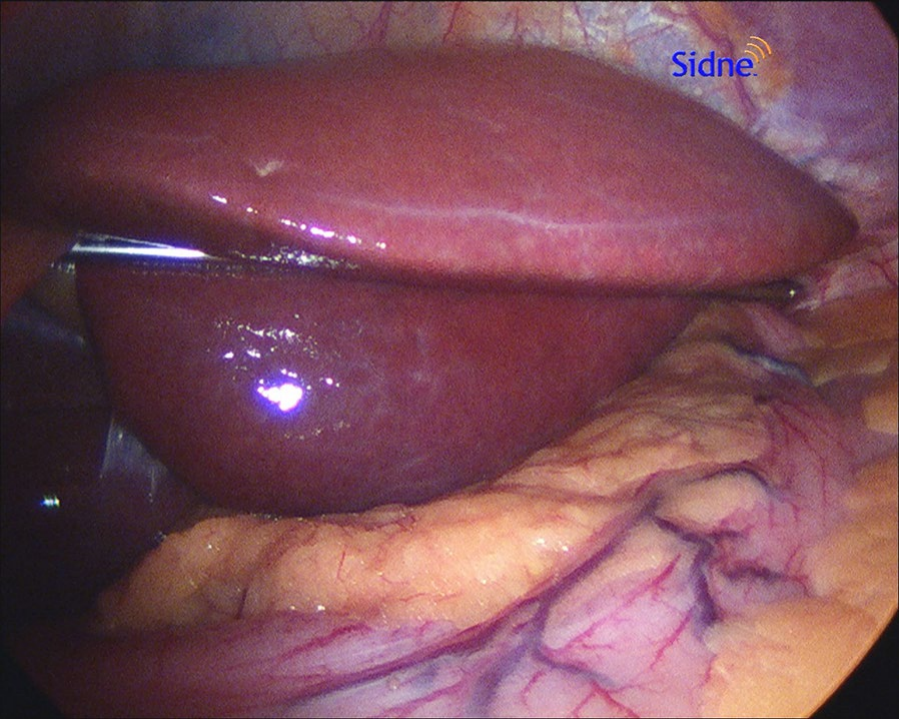
Technique of liver retraction with EndoLift device

Internal retraction is applied with a 2.0 silk stitch (30 cm) on a straight cutting needle (Keith) which is passed through the mid-upper abdomen 5–7 cm below the xiphoid process if the left lobe of the liver is found to be relatively small. The suture is picked up with non-toothed graspers and passed through the left lobe of the liver, about 5–7 cm medially from its edge. Then, it is passed back out proximal to the first insertion (through the abdominal wall) and gently pulled up to retract the liver. Needle insertion sites are monitored for any bleeding, bile leakage or laceration (Fig. 2). The alternative method utilizes the EndoLift Port-Free Retractor (Virtual Ports, Israel). It is comprised of a telescopic stainless steel bar positioned underneath the left liver lobe and two articulated clips on either end of the bar used to grasp and anchor it to the intra-abdominal wall (Fig. 3).

#### Single incision gastric bypass

We start the operation with creation of the jejuno-jejunal side-to-side stapled anastomosis, with a standard 50 cm biliopancreatic limb and 150 cm Roux limb. Our preferred site is the far upper-left of the peritoneal cavity and the placement of stay sutures is necessary. The closing of the resulting common enterotomy site with a stapler is difficult due to poor retraction. Therefore, we close the enterotomy site with the Endo-Stitch using a nonabsorbable suture.

The gastric pouch is created with the operative table in the steep reverse Trendelenburg position. We tend to create longer tubular shaped pouches to facilitate a low-tension anastomosis. A stitch is passed through the tip of the Roux limb to approximate it to the horizontal portion of the pouch. A 2 cm gastro-jejunal hand-sewn anastomosis is created in a two-layer fashion using absorbable suture applied with the Endo-Stitch. Sewing with the Endo-Stitch is difficult in this cranial position with only one grasper to guide the stitch and no counter-traction. We think it is important to pass the gastroscope prior to completion of the anastomosis to avoid strictures and to control bleeding. An air-leak test is always performed using a gastroscope.

## Results

A total of 14 morbid obese patients underwent SILS RYGB (n = 14) (twelve women and two men) with a mean age of 46 years. Mean weight at surgery was 113 kg (range 91–135). The mean BMI at the moment of surgery was 41.4 ± 5.3 kg/m^2^. The most prevalent preoperative co-morbidities in this selected group of patients included hypertension in 8 patients (57 %), hyperlipidemia in 6 (43 %), obstructive sleep apnea in 3 (21 %), hypothyroidism 3(21 %) and type 2 diabetes in 2 (14 %) (Table 1).

**Table 1:** Demographic characteristics

Variables	SILS- RYGB (n = 14)
Gender (F), n (%)	12 (86)
Age (years), mean ± SD	46 ± 11.1
BMI (kg/m^2^), mean ± SD	41.4 ± 5.3
Weight at surgery(kg), mean ± SD	113 ± 14.1
Most common comorbidities, n (%)	
Hypertension	8 (57)
Hyperlipidemia	5 (35.7)
Obstructive sleep apnea	3 (21.4)
T2DM	2 (14.3)

*SILS* single-incision laparoscopic surgery, *BMI* body max index, *T2DM* type 2 diabetes Mellitus

Intraoperative parameters showed a median operative time of 197 min (±40.4) and an estimated blood loss was 40 ml (range 20–100). In one patient, an intraoperative air leak was detected and fixed with over-sewing; this required placement of an additional 5-mm port. Overall, only 1 (7 %) patient required placement of one additional port and no conversions to conventional laparoscopic or open surgery was needed. In one patient, a narrowing at the gastro-jejunostomy was noted endoscopically. This required revision intraoperatively using the same single incision access without the need for additional ports. The median length of stay in the hospital was 3.4 days. Other 2 postoperative complications were postoperative hemoglobin drop necessitating blood transfusion, and a marginal ulcer. No patients required re-operation or readmission in the 90 days after surgery. No intraoperative or immediate postoperative deaths occurred (Table 2).

**Table 2:** Operative outcome

Variables	SILS- RYGB (n = 14)
Operative time (min), mean ± SD	197 ± 40.4
Estimated blood loss (cc), median (IQR)	40 (23.8-50)
90-day complication, n (%)	2 (14)
Conversion to conventional laparoscopic surgery or open surgery	0
Peri-operative complications, n (%)	
Leak test positive (added one port)	1 (7)
Intraoperative revision of GJ due to narrowing	1 (7)
Length of stay in hospital (days), median (IQR)	3.4 (3,4)
Return to work at one month, n (%)	6 (42.9)
Weight loss (lb.), mean ± SD	
First visit (7–10 days)	7.25 ± 4.5
Second visit (~1 month)	28.9 ± 11.86
4 months	45.4 ± 16.4

*SILS* single-incision laparoscopic surgery, *GJ* gastrojejunostomy

In terms of pain control, the mean frequency of PCA use was of 21 (range 35–4) times in POD0, 37 (range 59–6) times in POD1 and 13 times (range 99–0) in POD2. The pain score (VAS) was on POD0 (6.9 ± 2.2), POD1 (5.2 ± 1.9), and POD2 (3.9 ± 1.6). The frequency of oral pain medication and postoperative anti-emetic use is described in Table 3. 42.9 % of the patients returned to work in a 1 month period.

**Table 3:** Postoperative pain and nausea

Variables	SILS (n = 14)
PCA activation (frequency), median (IQR)	
POD 0	20.9 (9–26.5)
POD 1	36.6 (10–57.5.0)
POD 2	13.1 (1–25.5)
Pain severity (VAS score), mean ± SD	
POD 0	6.9 ± 2.2
POD 1	5.2 ± 1.9
POD 2	3.9 ± 1.6
Oral narcotic use	
In hospital (number of dosage), median (IQR)	2 (1–3)
After discharge (yes), n (%)	9 (64.3)
Anti-nausea medication (number of dosage), median (IQR)	
POD 1	3 (0-3)
POD 2	2 (0-2)

*SILS* single-incision laparoscopic surgery, *POD* postoperative day

**Fig. 4:**
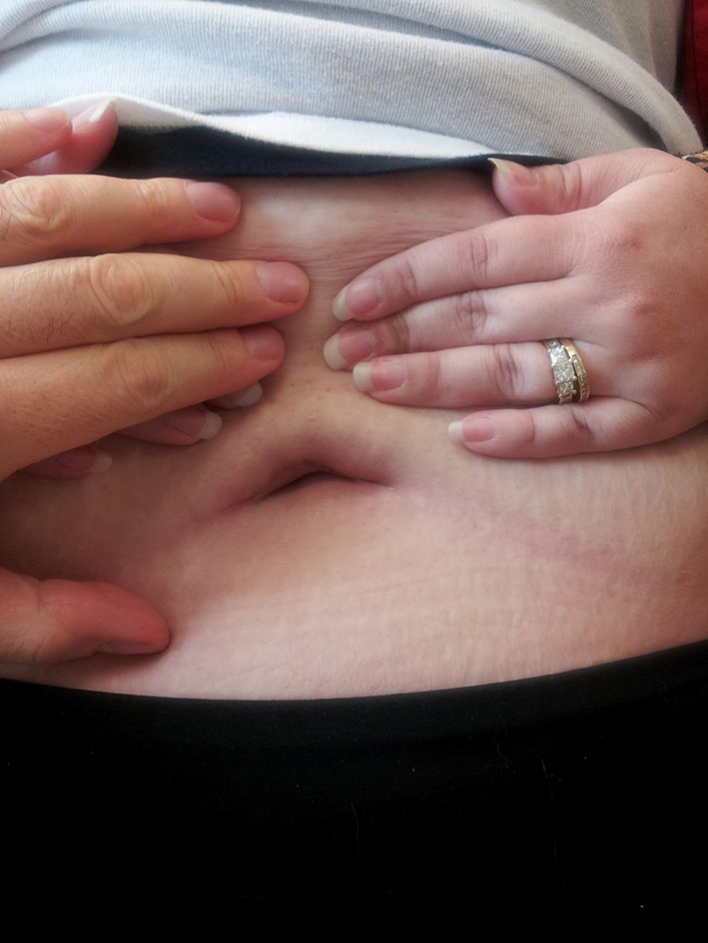
Cosmetic result

From a cosmetic point of view, patient satisfaction was high (all patients scored 3/3). Objective clinical assessment showed a well-hidden vertical scar measuring 2.5–3.5 cm within the umbilical niche (Fig. 4). No wound issues were noted postoperatively including wound infection, seroma, hernia, and dehiscence.

The short-term mean percent weight loss at 1 month was 28.9 lb. (±11.86) and after 4 months 45.4 (±16.4).

## Discussion

Laparoscopic surgery has now achieved a technical and operational maturity. This maturity has inspired an interest in even more minimally invasive procedures and to search ways to improve outcomes. Single-incision laparoscopic approaches are emerging in the field of bariatric and metabolic surgery. SILS not only may improve the patient’s aesthetic results but also can causes less damage to the abdominal wall, thereby causing less postoperative pain. However, there is no randomized trials corroborating this data.

Because the incision is transumbilical, the surgical scar is imbedded within the umbilicus and therefore, leaves almost no trace of the surgical access (Fig. 4). In our study, all the 14 patients scored a 3/3 in satisfaction regarding the surgical scar. Rogula et al. also showed better cosmesis satisfaction in SILS when compared to laparoscopic surgery [6], Lakdawala et al. demonstrated benefits in SILS sleeve gastrectomy regarding scar visibility [12].

Appropriate surgical candidate selection is very important to the success of single incision bariatric surgery. Most authors do not recommend SILS for patients with a BMI higher than 50 kg/m^2^. According to Mittermair et al., SILS is indicated predominantly for patients with a BMI of 35–45 kg/m^2^ [5]. In our study patients with BMI > 50 kg/m^2^a, a thick abdominal wall and tall stature were not considered for SILS. Also patients with scars from open surgery were not offered SILS due to questionable cosmetic benefits and expected adhesions.

A diversity of single incision and access modifications are described in literature. Ports can be placed either by introduction of multi-access port devices or conventional ports directly through separate fascial incisions. Huang K. et al. presented an omega shaped 4–6 cm incision, which is sometimes extended to almost a circular shape [7]. Varela et al. reports using multi-access port devices, which require a 3 cm incision above the umbilicus [8]. Fernandez et al. [9] and Merchant et al. [10] report using a gel-port device with three pre-installed trocars and one additional metal trocar for liver retraction. In our cases we preferred to access through vertical skin incision within the umbilicus for port placement. This allowed a subcutaneous platform for a broader placement of ports into the abdominal fascia. This approach seemed to be helpful especially for obese patients and provided better handling outside the patient’s body. Also, it eliminates the need for special devices or instruments, therefore reducing the potential risk if development of incisional hernia [11]. However, none of this different access techniques should compromise the patient’s safety or cosmetic outcome. Some studies have suggested that wound complications such as seroma, infection, and hernia are frequently mentioned as potential complications of SILS. For instance, Koh C. et al. demonstrated an 8.3 % superficial wound infection rate [11]. We had no apparent wound complications in our series after closing all fascial defects greater than 10 mm.

With regards to operative outcomes, two of our patients had intraoperative event that required redo of the gastro-jejunostomy anastomosis for leak or stricture. Huang reported no intraoperative complications in his SILS RYGB series. Our estimated blood loss, hospital stay and operative time was similar to other SILS studies.

Pain control was better after SILS (GB and SG) on POD 1 and POD 2, as measured by VAS pain score compared to conventional laparoscopic surgery in some studies [12]. However, no convincing trial with clinical benefit has been produced.

Our patients presented a weight loss of approximately 7.25 lb. (±4.5) after first postoperative visit, 28.9 lb. (±11.86) after 1 month and 45.4 lb. (±15.4) after 4 months.

Our methodology had some limitations. Retrospective design, small sample, short-term follow-up and single centered are limitations of this report.

## Conclusion

Single incision is feasible, safe and reproducible technique used as an access to complex surgeries like gastric bypass in carefully selected patients. Results in short-term outcomes are comparable to those observed in literature. Some potential benefits include less postoperative pain, improved cosmesis, and patient satisfaction. Randomized trials involving larger patient series with a longer follow-up and larger cohort studies and/or systematic reviews will be necessary to assess the extent of the benefits and limitations of SILS in bariatric surgery.

## References

[1] Reoch J, Mottillo S, Shimony A, et al. Safety of laparoscopic vs open bariatric surgery: A systematic review and meta-analysis. Arch Surg. 2011;146:1314–22. 10.1001/archsurg.2011.270 22106325

[2] Nguyen NT (2012). Strategic laparoscopic surgery for improved cosmesis in general and bariatric surgery: analysis of initial 127 cases. J Laparoendosc Adv Surg Tech A.

[3] Huang C-K, Lo C-H, Houng J-Y, Chen, Y-S, Lee P-H. Surgical results of single-incision transumbilical laparoscopic Roux-en-Y gastric bypass.Surg Obes Relat Dis. 2012;8:201–7. 10.1016/j.soard.2010.12.007 21296033

[4] Hawker GA, Mian S, Kendzerska T, French M. Measures of adult pain: Visual Analog Scale for Pain (VAS Pain), Numeric Rating Scale for Pain (NRS Pain), McGill Pain Questionnaire (MPQ), Short-Form McGill Pain Questionnaire (SF-MPQ), Chronic Pain Grade Scale (CPGS), Short Form-36 Bodily Pain Scale (SF-36 BPS), and Measure of Intermittent and Constant Osteoarthritis Pain (ICOAP). Arthritis Care Res (Hoboken). 2011;63 Suppl 11: S240–52. 10.1002/acr.20543 22588748

[5] Mittermair R, Pratschke J, Sucher R (2013). Single-incision laparoscopic sleeve gastrectomy. Am Surg.

[6] Rogula T (2014). Laparoscopic bariatric surgery can be performed through a single incision: a comparative study. Obes Surg.

[7] Huang C-K (2010). A novel surgical technique: single-incision transumbilical laparoscopic Roux-en-Y gastric bypass. Obes Surg.

[8] Varela JE (2009). Single-site laparoscopic sleeve gastrectomy: preclinical use of a novel multi-access port device. Surg Innov.

[9] Fernández JI, Ovalle C, Farias C, de la Maza J, Cabrera C (2013). Transumbilical laparoscopic Roux-en-Y gastric bypass with hand-sewn gastrojejunal anastomosis. Obes Surg.

[10] Merchant AM (2009). Transumbilical Gelport access technique for performing single incision laparoscopic surgery (SILS). J Gastrointest Surg..

[11] Koh CE, Martin DJ, Cavallucci DJ, Becerril-Martinez G, Taylor CJ (2011). On the road to single-site laparoscopic adjustable gastric banding: lessons learned from 60 cases. Surg Endosc.

[12] Lakdawala MA, Muda NH, Goel S, Bhasker A (2011). Single-incision sleeve gastrectomy versus conventional laparoscopic sleeve gastrectomy—a randomised pilot study. Obes Surg.

